# Association of atopy with disease severity in children with *Mycoplasma pneumoniae* pneumonia

**DOI:** 10.3389/fped.2023.1281479

**Published:** 2023-11-21

**Authors:** ChenRong Bian, SongTao Li, ShaoHu Huo, BeiBei Yang, PingPing Wang, WenHong Li, ShengGang Ding

**Affiliations:** ^1^ Department of Pediatrics, The First Affiliated Hospital of An Hui Medical University, Hefei, China; ^2^ Beijing Children’s Hospital, Capital Medical University, China National Clinical Research Center of Respiratory Diseases, Beijing, China

**Keywords:** children, *Mycoplasma pneumoniae* pneumonia, refractory *Mycoplasma pneumoniae* pneumonia, allergy, severity

## Abstract

**Background:**

*Mycoplasma pneumoniae* pneumonia (MPP) is common among children, but the impact of atopy on MPP severity in children is unknown. This study investigated whether atopic vs. nonatopic children had greater MPP severity.

**Methods:**

Retrospective analysis was conducted on 539 (ages 3–14 years) patients who were hospitalized in the First Affiliated Hospital of Anhui Medical University for MPP between January 2018 and December 2021, 195 were atopic and 344 were nonatopic. Of them, 204 had refractory MPP, and 335 had general MPP. And of atopic children, 94 had refractory MPP, and 101 had general MPP. Data on demographic and clinical characteristics, laboratory findings, clinical treatments were analyzed.

**Results:**

Significantly more boys with MPP were atopic than nonatopic (*P *< 0.05). More atopic (than nonatopic) children presented with prolonged fever and hospitalization, severe extra-pulmonary complications, asthma attaking, steroid and oxygen treatment, and increased IgE levels (all *P *< 0.05). In atopic (vs. nonatopic) children with MPP, the incidence of sputum plugs under the fiberoptic bronchoscopy and lobar pneumonia was significantly increased and required bronchoscopy-assisted and steroid therapy. Compared with nonatopic children, more atopic children developed refractory MPP (*P *< 0.05). Prolonged fever and hospitalization, severe extra-pulmonary complications, lymphocyte count, procalcitonin and lactate dehydrogenase levels, and percentages of atopy were all significantly higher (*P *< 0.05) among children with refractory MPP vs. general MPP. Moreover, Prolonged fever and hospitalization, lymphocyte count, procalcitonin and lactate dehydrogenase levels, and the treantment of steroid were all significantly higher (*P *< 0.05) among atopic children with refractory MPP vs. general MPP. Spearman correlation analysis showed strong associations between atopy and male sex, length of hospital stay, fever duration, IgE level, wheezing, lobar pneumonia, refractory MPP, and treatment with oxygen, hormones or bronchoscopy (*P *< 0.05).

**Conclusions:**

Atopy may be a risk factor for and was positively correlated with the severity of MPP in children.

## 1. Introduction

*Mycoplasma pneumoniae* is a common cause of community-acquired pneumonia, which is observed mainly in children and young adults, and it is an important and common pathogen of the respiratory tract. Most cases of *M. pneumoniae* pneumonia (MPP) are mild, but refractory MPP (RMPP) caused by *M. pneumoniae* infection is a serious disease with poor prognosis. The infection may lead to atelectasis, bronchiectasis, bronchiolitis obliterans, and necrotizing pneumonia and even involve other systems of the body, causing serious extra-pulmonary complications (1–3). Early prevention and treatment of RMPP has become an important issue for clinicians; however, the pathogenesis of RMPP remains unclear. A study by Lee et al. indicated that excessive host immune response mechanisms may be involved in the severity of MPP (4).

Atopy is considered a constitutional state in which the body is easily stimulated by the external environment and develops atopic diseases, such as atopic dermatitis, allergic rhinitis, and asthma. The population of people with allergic diseases is composed primarily of children with an atopic constitution. The main symptoms of an atopic constitution include skin itching, rash, nasal congestion, runny nose, sneezing, and cough, accompanied by CD4+ T helper type 2 cell differentiation and overproduction of immunoglobulin E (IgE) (5). Some studies have shown that an atopic constitution is a risk factor for recurrent lower respiratory tract infection, which may be closely related to a constitutional state associated with IgE antibodies and allergic diseases (6, 7). *M. pneumoniae* has strong clinical associations with asthma exacerbations and morbidity in both children and adults. Total and specific IgE responses have been described during *M. pneumoniae* respiratory infections. An increase in serum IgE levels in people with an allergic constitution can easily cause upper respiratory tract infection, and when excessive secretion cannot be discharged, it is transferred to the lower respiratory tract (7). Thus, there is a potential association between the reactive diseases caused by *M. pneumoniae* and atopy.

Several recent studies have reported that children with an atopic constitution are more likely to have a pulmonary *M. pneumoniae* infection (8). This infection can substantially increase IgE levels, especially during the acute phase and allergic state, suggesting that *M. pneumoniae* may mediate type I hypersensitivity during the development of MPP (9, 10). Although researchers have proposed that atopy may be a risk factor for the presence and severity of refractory MPP, no data have been available to indicate the effect of atopy on the severity of MPP. Total serum IgE levels in group of children with a high *M. pneumoniae* load were significantly higher than those in children with classic respiratory infections due to *M. pneumoniae* (8). These observations suggest that atopy may be associated with *M. pneumoniae*-related extra-respiratory manifestations. Specifically, *M. pneumoniae* infection induces an asthma attack by stimulating specific IgE, which can mediate a type I allergic reaction and eventually lead to tracheal spasm, airway hyper-responsiveness, and wheezing. The higher the lgE serum levels, the more frequent the wheezing attacks (11, 12), suggesting that *M. pneumoniae* and allergic diseases may have a similar immunological basis, at least in part. However, few studies have assessed the clinical characteristics of MPP in children with atopy and the severity of MPP among these children.

In this study, we investigated the clinical features, including respiratory disease severity, extra-respiratory manifestations, and total serum IgE levels, among children with MPP. The aim of this study was to explore the impact of atopy on the severity of MPP and to assess the role of IgE in disease pathogenesis.

## 2. Materials and methods

### 2.1. Study design and participants

A total of 539 children hospitalized with MPP at the First Affiliated Hospital of Anhui Medical University were consecutively enrolled in this study between January 1, 2018, and December 31, 2021. The ages of the children ranged from 3 to 14 years. Demographic data, laboratory findings, clinical characteristics, and treatments were recorded, and lung function and fibrobronchoscopy data were analyzed. This study was approved by the Ethics Committee of the First Affiliated Hospital of Anhui Medical University (Quick-PJ2023-09-29), and data from patients were collected anonymously.

To estimate the influence of atopy on respiratory severity, the children were divided into an atopic group and a nonatopic group. Children were additionally assigned to an RMPP group or a general MPP (GMPP) group to evaluate the impact of atopy on disease severity among children with MPP. Levels of IgE were compared between the atopic and nonatopic groups.

### 2.2. Diagnosis of MPP

Patients received a diagnosis of MPP based on their clinical presentation (e.g., fever, cough, dyspnea, and crackles), radiological findings, and changes in serum anti-mycoplasma antibody titers, tested using the microparticle agglutination method. Anti-mycoplasma IgM antibody titers with a ratio ≥1:160 during admission were considered positive, and IgG anti-mycoplasma titers more than fourfold higher in the symptomatic recovery phase than in the acute phase were considered positive (13, 14). Exclusion criteria were the follows (15): (1) had positive results for other pathogens; (2) had received corticosteroids, intravenous immunoglobulin (IVIG), and anticoagulation therapy before admission; (3) with underlying diseases, such as chronic cardiac and pulmonary disease, rheumatic diseases, and immunodeficiency; (4) had incomplete medical records.

### 2.3. Identification of atopy

We used a previous definition of atopy (16) as the presence of any one of the following conditions: (1) positive skin prick test or serum specific IgE; and (2) presence of allergic substances in the allergen 23 blood test used in our hospital.

### 2.4. Data collection

Nasopharyngeal aspirate or swab specimens were routinely tested within 24 h of admission. We collected data on demographic characteristics, imaging manifestations, and laboratory tests, including white blood cell and eosinophil counts and C-reactive protein (CRP), procalcitonin (PCT), lactate dehydrogenase (LDH), and total serum IgE levels. In addition, fiberoptic bronchoscopy, pulmonary function, and treatment regimens of all children infected with *M. pneumoniae* were retrospectively collected from medical records. Routine ventilation was used to collect respiratory data for children older than 6 years, with outcomes of forced vital capacity (FVC), forced expiratory volume in 1 s (FEV1), 1-s rate (FEV1/FVC), and peak expiratory flow (PEF). For children younger than 6 years, we used impulse oscillometry, collecting total impedance (Zrs), total airway resistance (R5), and reactance at 5 Hz (X5). During hospitalization, the patient's clinical signs and symptoms were recorded, including body temperature, living environment, and complications. Chest radiography was performed in all patients to confirm definite focal or segmental infiltrates and the presence of pleural effusion. Although patients had progressive symptoms, suspected complications, clinical deterioration, or persistent fever after appropriate antibiotic therapy, chest CT scans were performed. The large lesion was defined as the extent of infiltration on chest imaging more than one third of the lung. RMPP was diagnosed based on the presence of persistent fever, clinical or radiological deterioration after azithromycin treatment for 7 days or longer (1, 14), and one of the three can be diagnosed RMPP. The indications for bronchoscopy and glucocorticoid were evaluated according to the guidelines for management of community-acquired pneumonia in children (14).

### 2.5. Statistical analysis

The data were analyzed using SPSS version 22.0 software. Continuous variables were tested for normality using the Kolmogorov-Smirnov method and described using means and standard deviations or medians and interquartile ranges (IQRs). For normally distributed data, the difference between groups was tested using the independent samples *t*-test, and for non-normally distributed data, the Mann–Whitney *U*-test was used. Enumeration data were expressed as frequencies or percentages, and the differences between groups were tested using the *χ*^2^ test or the Fisher exact probability method. In this study, *P *< 0.05 using two-sided tests was considered statistically significant. Spearman correlation analysis was used to analyze whether there was correlation between two variables, and *P *< 0.05 was considered statistically significant.

## 3. Results

### 3.1. Comparison of clinical characteristics between atopic and nonatopic children with MPP

Of 539 enrolled children with MPP, 289 were boys and 250 were girls, with a mean age of 6.0 years (range, 3–14 years). Based on our definition of atopy, 195 (36.1%) children were assigned to the atopic group, and 344 children (63.9%) were assigned to the nonatopic group. There were significantly more boys in the atopic group (119; 61%) than in the nonatopic group (170; 49.6%) (*P *< 0.05). However, there was no significant difference in age or in the residential environment between the two groups (*P *> 0.05) (Table 1).

**Table 1:** Demographic and clinical characteristics of patients with MPP.

Characteristics	Atopic group (*n* = 195)	Nonatopic group (*n* = 344)	*P* value
Demographic data			
Age, years	6 (4, 7)	6 (4, 7)	0.706
Sex, no. (%)			
Male	119 (61.0%)	170 (49.4%)	0.012
Female	76 (39.0%)	174 (50.6%)	
Residence, no. (%)			
Urban	162 (83.1%)	266 (77.3%)	0.122
Rural	33 (16.9%)	78 (22.7%)	
Clinical characteristics			
Length of hospital stay, days	8 (6, 9)	7 (6, 9)	0.031
Duration of fever, days	7 (6, 9)	7 (6, 9)	0.044
Asthma attack, no. (%)	16 (8.2%)	9 (2.6%)	0.005
Complications, no. (%)	31 (15.9%)	32 (9.3%)	0.026
RMPP, no. (%)	94 (48.2%)	110 (32.0%)	<0.001
Type of pneumonia			
Lobar pneumonia, no. (%)	99 (50.8%)	126 (36.6%)	0.001
Bronchopneumonia, no. (%)	96 (49.2%)	218 (63.4%)	

Extra-pulmonary complications: multiple organ damage.

The length of hospital stay, duration of fever, and incidence of wheezing in the atopic group were significantly higher than in the nonatopic group (*P *< 0.05). Significantly greater percentages of patients with extra-pulmonary complications and RMPP were observed in the atopic group than in the nonatopic group (15.9% vs. 9.3% and 48.2% vs. 32.0%, respectively, all *P *< 0.05). In terms of imaging findings, the percentage of children with lobar pneumonia in the atopic group was significantly higher than that in nonatopic group (*P *< 0.05).

### 3.2. Comparison of laboratory characteristics, pulmonary function, and fiberoptic bronchoscopy results in atopic vs. nonatopic children

The atopic group had significantly higher total serum IgE levels than the nonatopic group, 217.5 [54.3–614.8] U/ml vs. 73.0 [30.5–164.5] U/ml; *P *< 0.01). There were no significant different in white blood cell counts (leukocytes, neutrophils, and lymphocytes) or CRP, PCT, or LDH levels between the two groups (all *P *> 0.05, Table 2). The results of fiberoptic bronchoscopy indicated that children with atopy more often had sputum plugs than children without atopy (*P *< 0.05). However, pulmonary function indexes (FEV1, FVC, FEV1/FVC, PEF, Zrs, R5, and X5) were not significantly different between the two groups (all *P *> 0.05).

**Table 2:** Laboratory, pulmonary function, and fiberoptic bronchoscopy findings of patients with MPP during admission.

** **	Atopic group (*n* = 195)	Nonatopic group (*n* = 344)	*P* value
WBC count (×10^9^/L)	6.6 (5.3, 8.0)	6.7 (5.3, 8.4)	0.659
Neutrophil count (×10^9^/L)	3.8 (2.9, 5.1)	4.0 (3.0, 5.2)	0.551
Lymphocyte count (×10^9^/L)	1.9 (1.5, 2.4)	2.0 (1.4, 2.6)	0.443
CRP, mg/L	14.9 (6.5, 24.3)	15.3 (7.1, 26.2)	0.310
LDH, U/L	321.5 (281.0, 364.8)	312.0 (280.2, 366.8)	0.643
Total IgE, U/ml	217.5 (54.3, 614.8)	73.0 (30.5, 164.5)	<0.001
PCT, ng/ml	0.0825 (0.05, 0.1)	0.095 (0.05, 0.2)	0.300
Pulmonary function			
FEV1, L	64.1 (57.0, 75.6)	71.7 (61.3, 75.6)	0.318
FVC, L	69.4 (60.8, 86.9)	71.8 (63.1, 85.1)	0.780
FEV1/FVC	92.1 (84.4, 101.0)	92.9 (84.5, 99.2)	0.888
PEF, L/s	58.0 (43.8, 68.0)	56.4 (45.2, 73.2)	0.772
Zrs, %	129.9 (98.1, 161.8)	118.8 (95.9, 141.0)	0.225
R5, %	124.4 (95.1, 163.5)	113.1 (97.8, 143.0)	0.466
X5, %	151.3 (109.5, 190.5)	100.0 (37.1, 174.6)	0.056
Fiberoptic bronchoscopy			
Sputum plugs, no. (%)	46 (61.3%)	42 (42.4%)	0.015

WBC, white blood cell; CRP, C-reactive protein; LDH, lactate dehydrogenase; IgE, immunoglobulin E; PCT, procalcitonin; FEV1, forced expiratory volume in 1 s; FVC, forced vital capacity; FEV1/FVC, 1-s rate; PEF, peak expiratory flow; Zrs, total impedance; R5, total airway resistance; X5, reactance.

### 3.3. Comparison of clinical treatments for children with vs. without atopy

As shown in Table 3, 218 children (40.4%) with MPP required hormone therapy, 47.2% in the atopic group vs. 36.6% in nonatopic group, a significant difference (*P *< 0.05). In addition, the use of oxygen therapy and fiberoptic bronchoscopy were significantly higher in the atopic group than in nonatopic group. By contrast, the days of hormone therapy was not significantly difference between the two groups (*P *> 0.05).

**Table 3:** Comparison of treatments for children with MPP by atopy group.

Treatment	Atopic group (*n* = 195)	Nonatopic group (*n* = 344)	*P* value
Oxygen treatment, no. (%)	20 (8.2%)	11 (2.6%)	0.001
Systemic steroid therapy, no. (%)	92 (47.2%)	126 (36.6%)	0.018
Systemic steroid therapy, days	3 (2, 4)	3 (2, 4)	0.978
Bronchoscope use, no. (%)	75 (38.5%)	99 (28.8%)	0.022

Steroid therapy: 2 mg/kg/day.

### 3.4. Clinical characteristics, laboratory findings, and atopy among patients by RMPP and GMPP group

As shown in Table 4, 204 children were assigned to the RMPP group, and 335 were assigned to the GMPP group. Compared with the GMPP group, the RMPP group exhibited a higher prevalence of atopy (*P *< 0.05). In addition, the RMPP group had a significantly higher length of hospital stay and duration of fever than the GMPP group (*P *< 0.05). There were significantly more severe extra-pulmonary complications in the RMPP group (*P *< 0.05), and lymphocyte counts, PCT levels, and LDH levels were significantly higher in the RMPP group than in the GMPP group (all *P *< 0.05). By contrast, there were no significant differences in age, sex, white blood cell counts, and CRP levels between the two groups (all *P *> 0.05).

**Table 4:** Clinical characteristics, laboratory findings, and atopy among children with MPP by RMPP and GMPP group.

** **	RMPP (*n* = 204)	GMPP (*n* = 335)	*P* value
Demographic data			
Age, years	6 (4, 7)	6 (4, 7)	0.070
Sex, no. (%)			
Male	110 (53.9%)	179 (53.4%)	0.929
Female	94 (46.1%)	156 (46.6%)	
Length of hospital stay, days	8 (7, 9)	7 (6, 8)	0.000
Duration of fever, days	9 (8, 10)	6 (5, 7)	0.000
Complications, no. (%)	31 (15.2%)	25 (7.5%)	0.006
Atopy, no. (%)	94 (46.1%)	101 (30.1%)	0.000
Laboratory findings			
WBC count (×109/L)	6.3 (5.3, 8.3)	6.7 (5.4, 8.3)	0.911
Neutrophil count (×109/L)	4.0 (2.9, 5.2)	3.9 (3.0, 5.0)	0.149
Lymphocyte count (×109/L)	1.9 (1.4, 2.3)	2.1 (1.6, 2.8)	0.002
CRP, mg/L	16.3 (9.3, 26.0)	14.9 (5.6, 24.5)	0.232
LDH, U/L	342.5 (290.5, 401.8)	306.0 (274.5, 357.0)	0.000
PCT, ng/ml	0.10 (0.06, 0.19)	0.08 (0.05, 0.15)	0.017

GMPP, general *Mycoplasma pneumoniae* pneumonia; RMPP, refractory *Mycoplasma pneumoniae* pneumonia; WBC, white blood cell; CRP, C-reactive protein; LDH, lactate dehydrogenase; IgE, immunoglobulin E; PCT, procalcitonin.

### 3.5. Clinical characteristics, laboratory findings, and treatments among atopic patients by RMPP and GMPP group

As shown in Table 5, 94 children were assigned to the RMPP group, and 101 were assigned to the GMPP group. The RMPP group had a significantly higher length of hospital stay and duration of fever than the GMPP group (*P *< 0.05). There were significantly more hormone treatment in the RMPP group (*P *< 0.05), and lymphocyte counts, and LDH levels were significantly higher in the RMPP group than in the GMPP group (all *P* < 0.05). By contrast, there were no significant differences in age, sex, extra-pulmonary complications, white blood cell counts, lymphocyte counts, PCT and CRP levels, length of hormone days and the use of bronchoscope between the two groups (all *P *> 0.05).

**Table 5:** Clinical characteristics, laboratory findings, and treatments among atopic children with MPP by RMPP and GMPP group.

** **	RMPP (*n* = 94)	GMPP (*n* = 101)	*P* value
Demographic data			
Age, years	7 (4, 8)	6 (3, 7)	0.088
Sex, no. (%)			
Male	56 (59.6%)	63 (62.4%)	0.769
Female	38 (40.4%)	38 (37.6%)	
Length of hospital stay, days	9 (8, 10)	7 (7, 9)	0.01
Duration of fever, days	9 (8, 10)	6 (6, 7)	0.000
Complications, no. (%)	14 (14.9%)	10 (9.9%)	0.289
Laboratory findings			
WBC count (×109/L)	7.0 (5.4, 8.5)	6.6 (4.7, 7.7)	0.733
Neutrophil count (×109/L)	4.4 (3.4, 5.4)	3.9 (2.8, 4.9)	0.202
Lymphocyte count (×109/L)	1.8 (1.3, 2.1)	1.9 (1.3, 2.7)	0.004
CRP, mg/L	18.8 (9.3,28.2)	14.3 (5.7, 28.9)	0.114
LDH, U/L	347 (285, 416)	329 (272, 387.0)	0.006
PCT, ng/ml	0.11 (0.07, 0.19)	0.11 (0.06, 0.16)	0.240
Treatment			
Systemic steroid therapy, days	3 (2, 5)	3 (2, 4)	0.261
Systemic steroid therapy, no. (%)	54 (57.4%)	38 (37.6%)	0.006
Bronchoscope use, no. (%)	39 (41.5%)	36 (35.6%)	0.402

GMPP, general *Mycoplasma pneumoniae* pneumonia; RMPP, refractory *Mycoplasma pneumoniae* pneumonia; WBC, white blood cell; CRP, C-reactive protein; LDH, lactate dehydrogenase; PCT, procalcitonin.

### 3.6. Binary logistic regression analysis between RMPP and GMPP

As shown in Table 6, it turned out that the atopy was the predictive factors for RMPP.

**Table 6:** Binary logistic regression analysis between RMPP and GMPP.

Variables	OR	95% CL	*P* value
Atopy	1.98	1.38–2.84	0.000

### 3.7. Correlations between atopy and several variables

Spearman correlation analysis was used to analyze the relationships between numerous variables and atopic constitution. As shown in Table 7, male sex, duration of fever, length of hospital stay, lobar pneumonia, asthma, systemic steroid therapy, bronchoscope-assisted treatment, and RMPP were strongly associated with atopy.

**Table 7:** Correlations between numerous variables and atopy.

** **	Male	Duration of fever	Length of hospital stay	Lobar pneumonia	Asthma attack	Systemic steroid therapy	Bronchoscope use	RMPP
Correlation coefficient	0.112	0.089	0.093	0.138	0.128	0.103	0.100	0.161
*P* value	0.009	0.038	0.031	0.001	0.003	0.160	0.021	0.000

RMPP, refractory *Mycoplasma pneumoniae* pneumonia.

## 4. Discussion

Mycoplasma is a class of microorganisms that fall between bacteria and viruses; they are without cell walls, highly polymorphic, and are 0.1–0.3 μm in size. *M. pneumoniae* can adhere to the ciliated epithelial cells of the respiratory tract after invading the human respiratory tract (17, 18), resulting in the production of cytokines and the recruitment of lymphocytes and other immune cells (19, 20). They may also spread outside the respiratory system to cause extra-respiratory manifestations in addition to respiratory diseases.

Infection with *M. pneumoniae* in children is a risk factor for developing allergic diseases and inducing allergy (21, 22). *M. pneumoniae* is an antigen, and after sensitization by this antigen, exposure to allergens induces IgE crosslinking, which triggers the degranulation and release of active substances, including enzymes, histamine, and cytokines that mediate the clinical manifestations of atopy (21). These cascade reactions also suggest that there is some relationship between *M. pneumoniae* and atopy. Several studies have shown that the clearance of *M. pneumoniae* in atopy is insufficient (22, 23). *M. pneumoniae* may elicit a T helper type 2 cell immune response in the bronchial system (17). In our study, total serum IgE levels were higher in atopic children than in nonatopic children. These results support previous findings that atopy is associated with an aberrant response to allergens through IgE production by antigen-specific T helper type 2 cells and B cells (24, 25). We also found that the prevalence of asthma attacks was significantly higher in atopic children than in nonatopic children, which suggested that atopic children with MPP have a high risk of asthma attack.

Our results also indicated that more children in the atopic group than in the nonatopic group developed refractory MPP and were likely to present with severe wheezing and prolonged fever and high incidence of lobar pneumonia and fiber bronchoscope-assisted therapy and hormone therapy. In addition, children in the RMPP group exhibited a higher prevalence of atopy. These findings indicated that the presence of atopy may be associated with severe MPP and with significantly greater difficulty in the successful management of this disease. Atopy may have an adverse impact on disease severity. Therefore, atopic patients with MPP should be treated more aggressively with macrolide antibiotics and glucocorticoids. *M. pneumoniae* as an antigen can induce an immune response, and the resultant inflammatory substances may lead to extra-respiratory manifestations in children with MPP, a finding in our study that is consistent with other studies (21). Some studies have reported that about 60% of children who are older than 5 years will develop asthma after first wheezing (26). Therefore, it is important to control wheezing in atopic children at an early stage. MPP has more severe clinical manifestations in atopic children, which may help to explain why children with atopy are more susceptible to other pathogens and have significantly higher disease severity than children without atopy.

IgE is an immunoglobulin that is present in small amounts but can cause type I hypersensitivity and a subsequent sensitized state of body when combined with basophils and mast cells. Some studies have also indicated that increased total serum IgE is an independent risk factor for asthma in children with wheezing (27), which can stimulate a series of immune responses in the body. Our study showed that the total serum IgE level was significantly higher in children with vs. without atopy, indicating that children are in a hypersensitive state after infection with *M. pneumoniae* (28), This finding is also consistent with the findings of a recent report (29) and shows the relationship between atopy and IgE production during *M. pneumoniae* infection. The increased IgE levels may be considered a marker of immune dysregulation (30, 31). Here, we showed that children with atopy are more prone to an immune response that includes increased IgE antibodies after exposure to *M. pneumoniae* (22).

Atopic children often have damage to epithelial cell barrier function and show decreased ciliary motion (32, 33), which creates local inflammation, enables easier colonization by *M. pneumoniae,* and makes it more difficult to clear the organisms from the respiratory tract after infection. Recent evidences supported the fact that *M. pneumoniae* is more than an extra-cellular pathogen colonizing epithelial cells of the respiratory tract. It is able to penetrate the cell membrane of host cells and to invade the respiratory mucosa, leading to pronounced inflammatory responses and also spreading outside the respiratory system, to some extent. Thus, direct and indirect (immune-mediated) mechanisms have been described in *M. pneumoniae* infections (34). Persistent chronic cough and rhinitis in children may increase the risk of airway infection. Some studies suggested glucocorticoid and fiberoptic bronchoscopy in children with mycoplasma pneumonia can effectively improve the clinical indicators of the children with promising efficacy and high safety (35–38). Therefore, we suggest that atopic children with MPP be provided with long-term hormone treatment as well as fiberoptic bronchoscopy—assisted treatment. Our bronchoscopy findings also indicated that many atopic children developed lymphatic follicular hyperplasia in the posterior pharyngeal wall. This is related to long-term nasal discharge backflow stimulation of the posterior pharyngeal wall in children with allergic rhinitis. These pathophysiological mechanisms highlight the need for atopic children to receive hormone and bronchoscopy-assisted treatments.

The present study has limitations. Our study was a single-center retrospective study, which may introduce selection bias; a multicenter, large-scale study is needed to confirm our findings. Previous findings have indicated significantly impaired small airway function and increased airway resistance among atopic children with MPP (39). By contrast, the present study found no significant differences in lung function between the atopic and nonatopic groups of children. However, not all hospitalized children in the present study had lung function examinations, leading to smaller sample size for these measures. Moreover, the relationship between immune-related factors and atopy in children with MPP was not explored further due to limited numbers of complete admission examinations at our center. Finally, in our study, we found that atopic patients infected with *M. pneumoniae* were more prone to have higher disease severity, but whether similar phenomena will be observed in atopic patients infected with other pathogens is unclear.

In conclusion, atopy may be a risk factor for MPP and its presence was positively correlated with disease severity in children with MPP. In addition, more severe clinical manifestation among children with RMPP may be related to bronchial mucosal inflammatory and concomitant immune response mechanisms. These findings may improve clinician understanding of MPP and inform treatment management of atopic children with MPP.

## Data Availability

The original contributions presented in the study are included in the article/Supplementary Material, further inquiries can be directed to the corresponding author.
